# Salt intake and salt‐reduction strategies in South Asia: From evidence to action

**DOI:** 10.1111/jch.14365

**Published:** 2021-09-09

**Authors:** Kamal Ghimire, Shiva Raj Mishra, Gautam Satheesh, Dinesh Neupane, Abhishek Sharma, Rajmohan Panda, Per Kallestrup, Craig S Mclachlan

**Affiliations:** ^1^ Nepal Development Society Bhratpur‐10 Chitwan Nepal; ^2^ School of Health Torrens University Sydney New South Wales Australia; ^3^ World Heart Federation Salim Yusuf Emerging Leaders Programme Geneva Switzerland; ^4^ The George Institute for Global Health Hyderabad Telangana India; ^5^ Welch Center for Prevention Epidemiology and Clinical Research Department of Epidemiology Johns Hopkins University Baltimore Maryland USA; ^6^ Department of Global Health Boston University School of Public Health Boston Massachusetts USA; ^7^ PRECISIONheor Precision Value & Health Boston Massachusetts USA; ^8^ Department of Research Public Health Foundation of India New Delhi India; ^9^ Department of Public Health Center for Global Health Aarhus University Aarhus Denmark

**Keywords:** cardiovascular disease, community‐based, dietary sodium‐intake, hypertension, salt reduction, South Asia

## Abstract

The World Health Organization recommends salt reduction as a cost‐effective intervention to prevent noncommunicable diseases. Salt‐reduction interventions are best tailored to the local context, taking into consideration the varying baseline salt‐intake levels, population's knowledge, attitude, and behaviors. Fundamental to reduction programs is the source of dietary salt‐intake. In South Asian countries, there is a paucity of such baseline evidence around factors that contribute to community salt intake. Upon reviewing the electronic literature databases and government websites through March 31, 2021, we summarized dietary salt intake levels and aimed to identify major sources of sodium in the diet. Information on the current salt reduction strategies in eight South Asian countries were summarized, namely Afghanistan, Bangladesh, Bhutan, India, Maldives, Nepal, Pakistan, and Sri Lanka. One hundred twelve publications (out of identified 640) met our inclusion‐exclusion criteria for full text review. Twenty‐one studies were included in the review. Quality of the included studies was assessed using the US National Heart, Lung, and Blood Institute assessment tool. The primary result revealed that mean salt intake of South Asian countries was approximately twice (10 g/day) compared to WHO recommended intake (< 5 g/day). The significant proportion of salt intake is derived from salt additions during cooking and/or discretionary use at table. In most South Asian countries, there is limited data on population sodium intake based on 24‐h urinary methods and sources of dietary salt in diet. While salt reduction initiatives have been proposed in these countries, they are yet to be fully implemented and evaluated. Proven salt reduction strategies in high‐income countries could possibly be replicated in South Asian countries; however, further community‐health promotion studies are necessary to test the effectiveness and scalability of those strategies in the local context.

## 1 INTRODUCTION

Globally, there are estimated 3 million deaths and 70 million disability‐adjusted life‐years (DALYs) attributable to high sodium intake (ie, primary dietary risk factor) in 2017.^1^ High sodium intake is associated with hypertension, that is, high blood pressure (BP), which is an important risk factor for cardiovascular diseases (CVDs) and stroke.^2^, ^3^, ^4^ Hypertension is reported as one of the three leading risk factors of the global burden of disease (GBD) attributing 7% of global DALYs.^5^ Hypertension accounts for 51% and 62% of global mortality due to coronary heart disease (CHD) and stroke, respectively.^2^ High salt intake also increases the risk of developing renal disease, osteoporosis, and gastric cancer.^6^ The increasing burden of hypertension—both in high‐income countries (HICs) and low‐ and middle‐income countries (LMICs)—is due to several factors embedded in individual lifestyle related behaviors, as well as health systems’ ability to control hypertension.^2^

The World Health Organization (WHO) recommends salt reduction as a “best buy” intervention; that is, a feasible and cost‐effective public health strategy to control noncommunicable diseases (NCDs) burden.^7^ The WHO's three pillars of sodium reduction include (i) product reformulation that entails reducing the salt content in commercialized foods and meals; (ii) raising consumers’ awareness on harmful effects of excessive salt intake, and about the benefits of reading food labels and of choosing healthier foods; (iii) developing an environment that conduces choosing the healthiest foods as an easy and affordable option for the population from all socioeconomic backgrounds.^8^ WHO recommends daily salt intake of less than 5 g (< 2 g sodium) for adults to control BP and reduce CVDs risk.^8^ Current estimates suggest the global mean intake of salt to be around 15.24 g (6 g sodium)/day in 2017 and higher intake estimates are found in Asian countries.^1^

South Asia (also regarded as SAARC ‐ The South Asian Association for Regional Cooperation) represents a culturally diverse and fast‐growing region and is home to a quarter of the world's population.^9^ Foods with high‐carbohydrate and low‐fat diets were considered a traditional dietary pattern of this region. However, over the last two decades, dietary patterns have changed due to globalization changes in food and agriculture. For example, increasing household‐income levels and cross‐border food trade have altered dietary patterns.^10^ Dietary transition from traditional diets (high plant‐based carbohydrate, fresh vegetables) to modern foods (low carbohydrate and high amount of animal‐based contents, sugar and processed foods) has also resulted in an epidemiological shift in regional diet induced diseases.^10^, ^11^ The growing burden of NCDs in the last decade—in addition to infectious diseases and nutrient deficiencies that were previously predominant—have resulted in a double burden of chronic diseases in South Asia.^12^ The trend of increasing population blood pressure and hypertension between the period of 1975 and 2015 resulted in not only HICs being the primary source but now to LMICs in South Asia and sub‐Saharan Africa.^13^ In 2015, 258 million (23%) of the 1.13 billion adults with raised blood pressure lived in South Asia (199 million in India).^13^ A systematic review and meta‐analysis reported a mean prevalence of hypertension of South Asian countries of 27% with variation in country level ranging from 13% to 16% in Bangladesh, 18‐48% in India, 15‐19% in Pakistan, 19‐27% in Sri Lanka, 22‐34% in Nepal, 24% in Bhutan, 32% in Maldives,^14^ and 29% in Afghanistan.^15^ High sodium intake was ranked as the leading dietary risk factor for premature death and disability in South Asia.^12^ The GBD study (2017) estimated salt intake for South Asia to be ≈ 10.16 g (≈ 4 g sodium)/day.^1^ A 2017 systematic review reported that salt intake in India ranged from 4.5 to 25.8 g/day with mean population intake to be 10.98 g/day.^16^

**FIGURE 1:**
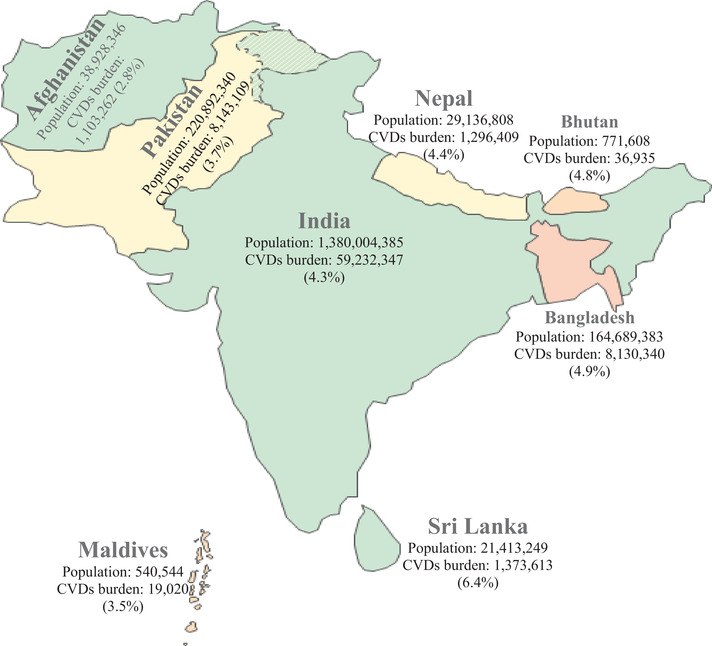
South Asia's regional map with population and prevalent cardiovascular disease in 2017^22^, ^23^

In 2013, the WHO Member States adopted the voluntary global target of a 30% reduction in mean population salt intake by 2025.^17^ In line with this target, the WHO South‐East Asia (WHO‐SEA) countries in 2013 set an intermediate, regional target of 10% reduction in mean salt intake over the next 5 years.^18^ To achieve the global salt reduction target, countries should tailor the interventions based on the respective population's current sodium intake level, knowledge, attitudes, and practices (KAP) related to salt intake, sources of dietary sodium in diet and determinants of high salt intake. However, there is scarcity of population‐based studies exploring these factors in most LMICs including in the South Asian countries. The burgeoning of multi‐morbidities and rising CVDs prevalence in South Asia^10^, ^19^, ^20^, ^21^ (Figure 1) calls for urgent and concerted actions among the member states to tackle the emerging risk factors including higher salt intakes. In this review, we present a narrative synthesis and analysis of existing evidence. The major objectives of this review are to (1) evaluate population level salt intake in available studies; (2) identify major sources of salt in the diet; (3) synthesize the current salt reduction policies, programs, and strategies in countries of South Asia namely Afghanistan, Bangladesh, Bhutan, India, Maldives, Nepal, Pakistan, and Sri Lanka.

## 2 METHODS

### 2.1 Search strategy and article selection

We searched for relevant literature in electronic databases PubMed/Medline and Cochrane Library along with Google Scholar. Search terms used were “sodium,” “sodium chloride,” “salt” in combination with “consume,” “intake,” “ingest,” “eat,” “diet,” “urine,” “excrete,” “hypertension,” “blood pressure,” “micronutrients,” “cardiovascular disease,” “stroke,” “South Asia,” “SAARC,” “Afghanistan,” “Bangladesh,” “Bhutan,” “India,” “Maldives,” “Nepal,” “Pakistan,” “Sri Lanka.” Reference lists of the selected articles were also cross‐checked. Additionally, we searched the World Bank, WHO and country government websites to locate the relevant information and documents. Local public health experts, researchers, and related non‐governmental organizations (NGOs) were also contacted to obtain salt related reports. Certain literature such as brief reports and conference proceedings were hand‐searched separately to obtain relevant data (supplementary file 1).

### 2.2 Inclusion and exclusion criteria

Studies were included if they reported estimates of salt/sodium intake, dietary sources of sodium in South Asian countries regardless of sample size. We limited studies to those published in English language through March 31, 2021. Studies that did not report standard statistics (mean/median intake of salt) and stated only percentage of high salt intake were excluded.^24^

### 2.3 Data extraction, synthesis, and analysis

For each South Asian country, we extracted information pertaining to salt intake, including sample size, age and sex of the study population, methods of salt intake measurement, sources, and amount of dietary salt intake. Estimates of sodium intake, if measured based on diet or urinary samples, were converted to mass of sodium chloride (g/day) using standard conversion values, that is, 1 g sodium chloride = 17.1 mmol or 393.4 mg sodium.^25^ Mean salt intake from the included studies was obtained; however, given the heterogeneity in methods of salt assessment, these estimates were not pooled further. Salt reduction initiatives including policies, programs, and strategies in South Asian countries were categorized based on the WHO's three pillars of intervention.^8^ Two authors (KG and SG) independently conducted preliminary title and abstract screening based on inclusion‐criteria and extracted relevant data from the studies selected for full‐text screening. Any discrepancies in review and data extraction were resolved through a consensus discussion with a third author (SRM).

## 3 RESULTS

### 3.1 Selected studies

**FIGURE 2:**
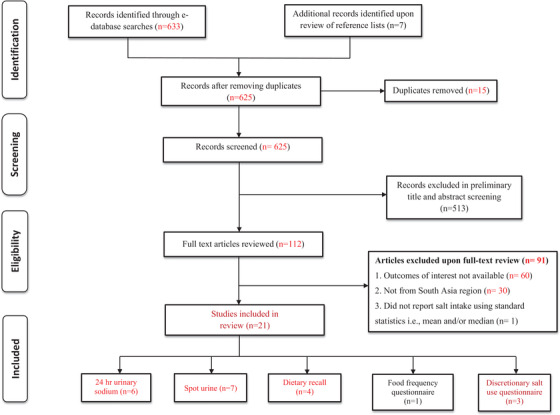
PRISMA flow‐chart for systematic review of studies

A total of 633 search results were identified through the database search and seven additional articles were identified by cross‐checking of reference lists (total 640 records). After removing 15 duplicate records, 625 studies were double screened by two authors (KG and SG). Of the 625 records, 513 were excluded based on the abstract and title ineligibility. Remaining 112 records were given full text assessment in which 21 studies met the eligibility criteria and were included in the review (Figure 2).

### 3.2 Characteristics of included studies

Of the 21 included studies,^3^, ^15^, ^26^, ^27^, ^28^, ^29^, ^30^, ^31^, ^32^, ^33^, ^34^, ^35^, ^36^, ^37^, ^38^, ^39^, ^40^, ^41^, ^42^, ^43^, ^44^ a third of the studies (n = 7) were from India, followed by Nepal (n = 5), Bangladesh (n = 4), and Sri Lanka (n = 2). Afghanistan, Bhutan, and Pakistan each had one study. Our search did not yield any studies and documents related to salt/sodium intake and sources from Maldives. Among the 21 studies, 62% used urinary excretion methods to estimate sodium intake [24‐h (n = 6), spot urine (n = 7)]. Remaining eight studies used dietary survey methods [dietary recall (n = 4), discretionary salt use questionnaire (n = 3), food frequency questionnaire (n = 1)]. We found only two studies from India that also measured dietary sources of sodium in diet.^31^, ^34^ (Table 1, Figure 2)

**TABLE 1:** Characteristics of studies that examined mean salt intake and sources

Country	Study and year	Sample size, age, and sex examined	Methods of measuring sodium intake	Sodium intake estimated	Dietary sources of sodium measured
Afghanistan	STEPS (2018)^15^	A national representative STEPS survey involving 3956 individuals (aged 18–69); 48.8% female	Estimated 24‐hour sodium excretion using spot urine samples (INTERSALT equation without potassium)	Yes	No
Bangladesh	Ahsan and associates (2020)^26^	131 respondents aged 54.3±14.4 years; 56.48% female	Questionnaire on discretionary salt use in cooking or at the table	Yes	No
Bangladesh	Zaman and associates (2017)^27^	200 (100 rural and 100 urban) respondents aged ≥20 years; 50% female	Estimated 24‐h sodium excretion using spot urine samples (Tanaka equation) and, Questionnaire on discretionary salt use in cooking or at the table	Yes	No
Bangladesh	Rasheed and associates (2014)^28^	388 participants aged 25–105 years (mean age: 44.6 years); 48.96% female	24‐h urinary sodium excretion	Yes	No
Bangladesh	STEPS (2018)^29^	A national representative STEPS survey involving 8185 individuals (aged 18–69); 53.5% female	Estimated 24‐h sodium excretion using spot urine samples (Tanaka equation)	Yes	No
Bhutan	STEPS (2020)^30^	A national representative STEPS survey involving 5575 individuals (aged 15–69); 61.3% female	Estimated 24‐h sodium excretion using spot urine samples (INTERSALT equation without potassium)	Yes	No
India	Johnson and associates (2019)^31^	1283 participants from urban and rural areas of North and South India; mean age 40.1 years; 48.2% female	24‐h dietary recall	Yes	Yes
India	Mathur and associates (2021)^32^	A national representative cross‐sectional survey involving 10659 individuals (aged 18–69 years with mean age of 40.1±13.8 years); 54.6% female	Estimated 24‐h sodium excretion using spot urine samples (INTERSALT equation with potassium)	Yes	No
India	Johnson and associates (2017)^33^	1395 participants from urban and rural areas of North and South India; mean age 40.2 years; 48.2% female	24‐hurinary sodium excretion	Yes	No
India	Ravi and associates (2016)^34^	6876 individuals (aged 42.62±9.93 years); 56.89% female	24‐h dietary recall	Yes	Yes
India	Kumbla and associates (2016)^35^	446 participants (aged 55.3±10.43 years); 43.04% female	Three‐day recall of food item and, Three‐day food diary	Yes	No
India	Radhika and associates (2007)^36^	1902 participants [aged 36.7±11.2 years (normotensive) and 44.9±12.9 years (newly diagnosed)]; 56.83% female	Food frequency questionnaire and, Questionnaire on discretionary salt use in cooking or at table	Yes	No
India	INTERSALT (1988)^3^	399 participants from two sites of India (Ladakh: 200 and New Delhi: 199)	24‐h urinary sodium excretion	Yes	No
Nepal	Neupane and associates (2020)^37^	451 participants aged 49.6±9.8 years; 65.41% females	24‐h urinary sodium excretion	Yes	No
Nepal	Dhimal and associates (2020)^38^	A national representative STEPS survey involving 5593 individuals (aged 15–69); 63.3% female	Estimated 24‐h sodium excretion using spot urine samples (INTERSALT equation without potassium)	Yes	No
Nepal	Ghimire and associates (2019)^39^	2815 participants aged 45.2±10.2; 65.47% female	Questionnaire on discretionary salt use in cooking or at the table	Yes	Partly
Nepal	Dhungana and associates (2014)^40^	406 participants aged 36.2±9 years; 56.65% female	Questionnaire on discretionary salt use in cooking or at the table	Yes	No
Nepal	Kawasaki and associates (1993)^41^	927 participants of two representative hilly and suburban villages of Nepal (aged 38.9±1.4); 49.1% female	Estimated 24‐h sodium excretion using spot urine samples (Kawasaki equation)	Yes	No
Pakistan	Saqib and associates (2020)^42^	Participants, mean age 26.5±5 years, 23% females	24‐h urinary sodium Spot urine method	Yes	No
Sri Lanka	Gamage and associates (2017)^43^	167 participants aged 30–60 years (mean age: 46.56±7.9); 68.9% female	24‐h urinary sodium excretion	Yes	No
Sri Lanka	Jayawardena and associates (2014)^44^	463 participants, aged ≥18 years; 64.14% females	24‐h dietary recall	Yes	No

INTERSALT, International Study of Salt and Blood Pressure; STEPS, WHO STEPwise approach to non‐communicable disease risk factor surveillance.

### 3.3 Quality assessment of included studies

Quality assessment and risk of bias in studies were assessed using validated checklists published by the US National Heart, Lung, and Blood Institute (NHLBI).^45^ These checklists have been designed to evaluate internal validity and bias risk for cross‐sectional studies and included 14 criteria. The criteria were rated as either yes, no, or “other” (ie, CD, cannot determine; NA, not applicable; NR, not reported).^45^ The overall assessment of the studies was classified as “good,” “fair,” or “poor” based on the percentage of “yes” calculated. Studies with lower than 50% “yes” score were considered poor, those with 50–75% were considered fair, and those with > 75% were considered good quality.^45^ Criteria that were not applicable (such as repeated exposure assessment, loss to follow‐up after baseline in case of cross‐sectional studies) were not considered in our quality assessment. Quality assessment was done independently by two authors (KG and SG) and finalized upon discussion with third author (SRM). Of the 21 included studies, half (n = 11) were of intermediate quality (fair) followed by six studies, rated as good and remaining four studies were of poor quality. According to NHLBI guideline, criterion‐6 (exposure assessed prior to outcome measurement) and criterion‐7 (sufficient timeframe to see an effect) would get a “no” response in case of cross‐sectional studies.^45^ Quality assessment revealed lack of measuring and/or reporting of participation rate of eligible persons, sample size justification (power description or variance or effect estimates) and blinding of outcome assessors to the exposure status. Details of quality assessment are shown in supplementary file 2.

### 3.4 Dietary salt intake in available studies of South Asia

**FIGURE 3:**
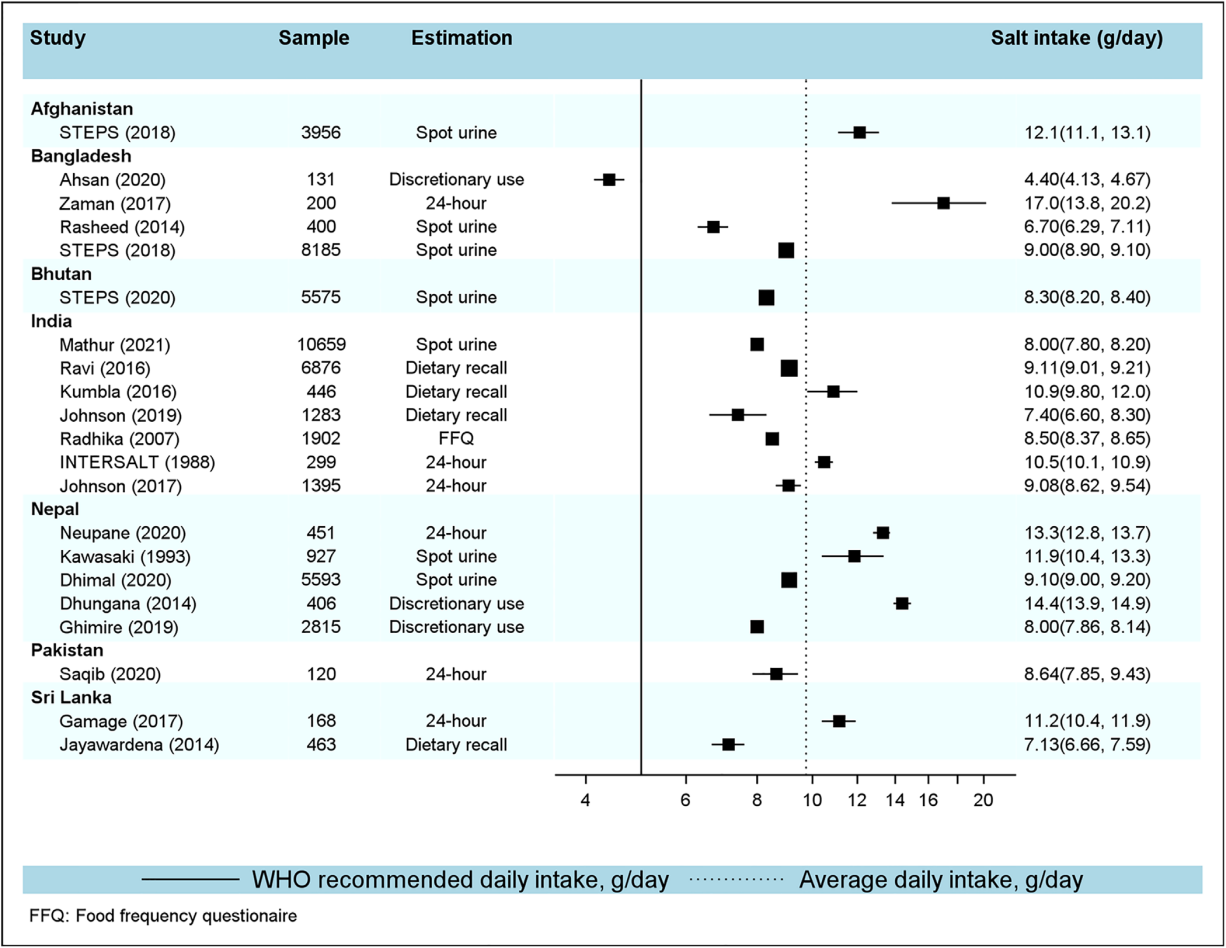
Forest plot showing salt intakes in South Asia

In our review, we found that varied levels of salt intake have been reported in South Asia, which can partly be attributed to the differences in measurement methods and/or dietary practices. Salt intake in included studies ranged from 4.4 (4.13‐4.67)g/day^26^ to 17.0 (13.8‐20.2)g/day^27^ and the highest intake was reported from Bangladesh.^27^ Data revealed that salt intake in all South Asian countries is higher than WHO recommendation level of < 5 g/day. Though the mean salt intake appeared to be approximately 10 g (9.7 g)/day, these estimates were not pooled further due to heterogeneity in measuring sodium intake across studies. Details of salt intake, sample size, study design, and measurement methods are presented in Tables 1 and 2, and in Figure 3.

**TABLE 2:** Levels of salt intake in South Asian countries

		Levels of salt intake (estimate, 95% CI)/g/day		
Country	Study and year	Male	Female	Both sexes
Afghanistan	STEPS (2018)^15^	12.5 (10.9 ‐ 14.0)	11.8 (10.5 ‐ 13.1)	12.1 (11.1 ‐ 13.1)
Bangladesh	Ahsan and associates (2020)^26^	–	–	4.4 (4.13 ‐ 4.67)
Bangladesh	Zaman and associates (2017)^27^	17.6 (14.0 ‐ 21.2)	16.3 (12.4 ‐ 20.2)	‐ Urine sample: 17.0 (13.8 ‐ 20.2) ‐ Dietary questionnaire: 13.4 (7.3 ‐ 19.5)
Bangladesh	Rasheed and associates (2014)^28^	–	–	6.7 (6.29 ‐ 7.11)
Bangladesh	STEPS (2018)^29^	9.0 (8.9 ‐ 9.1)	9.0 (8.9 ‐ 9.2)	9.0 (8.9 ‐ 9.1)
Bhutan	STEPS (2020)^30^	9.1 (8.9 ‐ 9.2)	7.4 (7.3 ‐ 7.5)	8.3 (8.2 ‐ 8.4)
India	Johnson and associates (2019)^31^	–	–	7.40 (6.60 ‐ 8.30) ‐ Andhra Pradesh: 8.72 (7.62 ‐ 9.81) ‐ Delhi and Haryana: 5.62 (5.24 ‐ 6.0)
India	Mathur and associates (2021)^32^	8.9 (8.7 ‐ 9.2)	7.1 (6.9 ‐ 7.2)	8.0 (7.8 ‐ 8.2)
India	Johnson and associates (2017)^33^	9.73 (9.08 ‐ 10.39)	8.33 (7.7 ‐ 8.96)	9.08 (8.62 ‐ 9.54) ‐ Andhra Pradesh: 9.46 (9.06 ‐ 9.85) ‐ Delhi and Haryana: 8.59 (7.68 ‐ 9.51)
India	Ravi and associates (2016)^34^	10.4 (10.3 ‐ 10.5)	8.13 (8.03 ‐ 8.23)	9.11 (9.01 ‐ 9.21)
India	Kumbla and associates (2016)^35^	–	–	10.9 (9.8 ‐ 12.0)
India	Radhika and associates (2007)^36^	–	–	8.5 (8.37 ‐ 8.65)
India	INTERSALT (1988)^3^	–	–	10.48 (10.1 ‐ 10.86) Ladakh: 11.9 (11.29 ‐ 12.5) New Delhi: 9.38 (8.90 ‐ 9.85)
Nepal	Neupane and associates (2020)^37^	14.4 (13.6 ‐ 15.2)	12.7 (12.2 ‐ 13.2)	13.3 (12.8 ‐ 13.7)
Nepal	Dhimal and associates (2020)^38^	9.6 (9.4 ‐ 9.8)	8.7 (8.6 ‐ 8.8)	9.1 (9.0 ‐ 9.2)
Nepal	Ghimire and associates (2019)^39^	–	–	8.0 (7.86 ‐ 8.14)
Nepal	Dhungana and associates (2014)^40^	–	–	14.4 (13.9 ‐ 14.9)
Nepal	Kawasaki and associates (1993)^41^	12.53 (7.70 ‐ 17.35)	11.18 (4.87 ‐ 17.49)	11.85 (10.38 ‐ 13.33)
Pakistan	Saqib and associates (2020)^42^	9.23 (8.42 ‐ 10.0)	6.54 (5.91 ‐ 7.17)	24‐h urine 8.64 (7.85 ‐ 9.43) Spot urine 7.82 (7.15 ‐ 8.49)
Sri Lanka	Gamage et al., (2017)^43^	–	–	11.15 (10.4 ‐ 11.9)
Sri Lanka	Jayawardena and associates (2014)^44^	8.28	6.37	7.13 (6.66 ‐ 7.59)

CI, confidence interval; INTERSALT, International Study of Salt and Blood Pressure; SD, standard deviation; STEPS, WHO STEPwise approach to non‐communicable disease risk factor surveillance.

### 3.5 Recurring themes of salt sources in the diet

Capturing information on dietary sources of salt/sodium will enable countries to develop culturally tailored salt‐reduction programs. Dietary surveys (food consumption database) and food composition databases are the commonly recommended methods to assess sources of sodium at the population level.^8^ In dietary surveys, food consumption of the entire population (in stratified sampling and population subgroups) is captured, and precise information of food consumption patterns is generated. Some of the tools are 24‐h recall of foods consumed (considered gold standard method), food frequency, food diaries, weighed food method, and retail sales data (by market research companies). Food composition databases may consist of nutritional information of commonly consumed food at either the local, national or regional level, and can be updated through various sources such as scientific literature, corporate websites, food industry, and labeling information.^8^

Sources of dietary sodium differ between HICs and LMICs. While manufactured and processed foods occupy 80% of the salt intake in HICs, the majority of consumed salt in LMICs is added during food preparation (cooking) and/or discretionary table salt.^46^, ^47^ Recent data demonstrate that salt use while cooking or at the table constitute 98% both in Nepal^48^ and in Afghanistan,^15^ 83% (in Delhi and Haryana) and 88% (in Andhra Pradesh) in India,^31^ 80% in Bangladesh,^49^ 53% in Sri Lanka^50^ and 42% in Bhutan,^51^ of total dietary salt intake.

Data are not available for other food sources of sodium intake in most South Asian countries. A study conducted in India reported that main food sources of sodium intake varied by in‐country regions.^31^ In India's Andhra Pradesh region, these sources were meat, poultry, and eggs (6.33%), dairy and dairy products (2.62%), fish and seafood (1.61%), and fruits and vegetables (1.11%), and in (ii) Delhi‐Haryana region, these were dairy and dairy products (6.36%), bread and bakery products (3.34%), fruits and vegetables (2.07%) and snack foods (2.05%).^31^ Another study conducted in South India identified key food sources of sodium were pulses (29.7%), rice‐based dishes (27.03%), vegetables (16.7%), fruits (7.8%), milk and dairy (4.07%), green leafy vegetables (3.20%), roots and tubers (2.87%), wheat flour and products (2.20%), nuts and oil seeds (1.71%) and other grains (1.35%).^34^ Consumption of processed foods is still relatively low in South Asian countries; therefore, the marked effect of nutritional transition is yet to be widely observed.^31^, ^34^ It is expected that the transition period in food choice will also see an increase in individual salt intake.

### 3.6 Salt reduction strategies, and effects on blood pressure and cardiovascular diseases

The relationship between sodium intake and BP has been extensively studied.^3^, ^52^, ^53^, ^54^ The International Study of Salt and Blood Pressure (INTERSALT study) examined a significant positive relationship between salt intake and BP, and estimated that 6 g/day increment in salt intake is associated with 9 mmHg elevation in systolic BP over a 30‐year period.^3^ High BP and elevated risk of CVDs and stroke impose both clinical and societal burden; not only do CVDs result in disability and mortality, but also add economic burden for the individual, community and health system. These burdens can be avertable by simply reducing salt intake. Estimates suggest that the need for antihypertensive treatment would decrease by half and deaths due to cerebrovascular diseases and CHD would decline by 22% and 16%, respectively, if universal reduction of sodium intake of 50 mmol/day (equivalent to 1.2 g salt/day) can be achieved.^56^ Government interventions targeting a 10% reduction of population salt intake over 10 years were projected to be highly cost‐effective worldwide and expected to avert approximately 5.8 million CVDs‐related DALYs/year.^55^ The population mean cost‐effectiveness ratio was estimated to be approximately international dollars (I$) 204/DALY.^55^

Community‐based salt reduction programs have been conducted in many parts of the world, resulting in reduced salt intake, increased awareness and lowering of BP.^57^, ^58^, ^59^, ^60^, ^61^ Community‐based Eat Less Salt intervention of Vietnam was able to decrease community salt intake from 21.53  to 20.44 g/day together with reduction in systolic blood pressure by 5.93 mmHg and diastolic blood pressure by 4.86 mmHg between baseline and follow‐up of 1‐year intervention.^60^ Similar project in Australia demonstrated a mean salt intake reduction from 8.8  to 8 g/day and increased in salt related KAP over a 3‐year period.^61^ Studies conducted in Japan and Portugal have found significant reduction in community salt intake, and a corresponding reduction in BP.^62^ Similarly, in Finland, population mean salt intake decreased from 12 g/day to < 9 g/day between the year 1979 to 2002 and a BP reduction of > 10 mmHg was noted.^62^ Salt reduction in Finland also resulted in 75%‐80% decline of both stroke and CHD related death ‐ this corresponded to increased life expectancy by 5–6 years over the period of three decades.^62^ A meta‐analysis of randomized controlled trials showed that a moderate reduction in salt intake of 4.4 g/day resulted in lower BP both in normotensive and hypertensive people (2/1  and 5/3 mmHg, respectively).^58^ The meta‐analysis concluded that higher sodium reduction would result in a possibly higher BP reduction.^58^ The Dietary Approaches to Stop Hypertension (DASH) study found that a salt reduction program in combination with healthy diet had a synergistic effect on reducing systolic BP.^59^ This was accompanied by a significant reduction in those who eat lower sodium containing foods compared to those who eat foods with higher sodium levels.^59^

Existing evidence suggest high‐cost savings from implementation of population level salt reduction initiatives. A Canadian study has estimated that a decrease in salt intake of the order of 4.6 g/day could result in nearly one‐third reduction of hypertension prevalence, which in turn could result in annual health savings of USD 430 million.^63^ Similarly, a Norwegian study estimated that the decrease of mass salt to 6 g/day could lead to 2 mmHg systolic BP reduction, which in turn would result in USD 4.7 million cost savings annually.^62^

Global estimates suggest that across all regions, salt reduction intervention was found best cost‐effectiveness in South Asia, projecting to avert 1.13 million CVDs related DALY/year with estimated mean cost of I$116/DALY over 10 years.^55^ The corresponding data for India was estimated to be < USD100/DALY^64^ and projected to save 2 million lives in 10 years.^18^ It is estimated that 1–3 mmHg reduction in systolic BP can be achieved at the population level by a 15% reduction in population salt intake in the WHO SEA region in 10 years.^18^ There is, however, an absence of operational research and evidence on cost‐effectiveness and potential economic impact of salt reduction programs at the national and regional level.^18^ Successful community‐based salt reduction programs and strategies implemented in HICs can potentially be replicated in South Asia with culturally tailored interventions in the local context. Some of the strategies are: (i) partnerships with the food industry for salt limitation in manufactured foods ‐ voluntary and mandatory processes and reformulation of processed foods that are high in salt and account for a large percentage of intake; (ii) health education and awareness activities regarding harmful effects of high salt intake; (iii) mandatory customer‐friendly labeling of sodium contents in processed foods; (iv) increased accessibility and availability of low salted healthy foods to customers for easier dietary choices.^65^

### 3.7 Salt reduction policies, programs, and strategies in South Asia

Many of the salt reduction plans, programs, and strategies in South Asian countries are yet to be fully implemented and evaluated. Hereby, we outline the current salt reduction initiatives in individual South Asian countries (Table 3).

**TABLE 3:** Summary of salt reduction initiatives in South Asian countries classified according to the WHO's three pillars of intervention

	WHO's pillars for salt reduction strategies		
Countries	Product reformulation	Consumer awareness	Environmental change
Afghanistan^66^, ^67^	‐Plans to reformulation of food products to decrease salt.	‐Developed food ‐based dietary guidelines in 2015. ‐Plans to conduct mass media campaign to reduce salt intake.	NA
Bangladesh^68^, ^69^	‐Plans to reformulate food products to decrease salt.	‐Developed dietary guidelines in 2000 and revised in 2013. ‐Plans to implement national salt reduction campaigns in mass media, schools, and institutions.	‐Plans for promotion of nutritional labelling for all pre‐packaged foods. ‐Plans to discourage sale of processed foods high in salt in schools and workplace catering facilities.
Bhutan^70^, ^71^	‐Plans to collaborate with food industries to limit the salt contents in processed foods and restriction in importing high salted processed foods.	‐Introduced behaviour change communication and public health campaigning of low salt intake. ‐Developed food‐based dietary guideline in 2011.	‐Plans to establish guidelines for nutritional labelling for all pre‐packaged foods. ‐Regulate the identified unhealthy food high in salt from school and workplace premises.
India^73^, ^75^, ^96^	‐Plans to regulate reformulation of processed foods with reduces salt content and expand the scope of Food & Nutrition program to develop better technologies for food reformulation.	‐Proposed strategies of public health campaigning to increase consumer awareness about the harmful effect of high salt intake. ‐Developed dietary guidelines in 1998 and revised in 2011.	‐Initiated mandatory display of red‐color coding on their labels for food products that are high on salt content levels. ‐Plans to regulate in marketing and advertisement of foods high in salt. ‐Promote low salted healthy food in trains and at railway stations. ‐Proposed to impose highest GST for foods high in salt.
Maldives^76^, ^77^	‐Plans to regulate private industry to voluntarily reduce salt in packaged food and monitor compliance.	‐Developed national food‐based dietary guidelines in 2018. ‐Proposed policies to reduce food marketing and advertisement of foods high in salt. ‐Plans to conduct public campaigns through mass media and social media to encourage consumers to eat less salt.	‐Plans to ban foods high in salt from school premises and workplace catering facilities. ‐Plans for promotion of nutritional labelling for all pre‐packaged foods.
Nepal^78^	‐Plans to regulate salt content reduction in packaged food and monitor compliance.	‐Plans to develop advocacy and awareness program to educate people on low salt use through various mass media. ‐Developed food‐based dietary guidelines in 2004 and revised in 2012.	‐Plans to ban foods high in salt from school premises and workplace catering facilities.
Pakistan^66^, ^80^	NA	‐Developed food‐based dietary guidelines for better nutrition in 2018 which set the recommended daily salt intake of < 5 g/day.	‐Plans to develop guideline for nutrition labelling.
Sri Lanka^71^, ^81^, ^83^	‐Plans to establish a mechanism to ensure voluntary and mandatory reduction of salt. ‐Motivate the food industry, food processors and food retailers to reformulate processed foods.	‐Developed food‐based dietary guideline in 2002 and revised in 2011 and in 2016. ‐Introduced behavior change communication and public health campaigning of low salt intake. ‐Plans to publish commercials on salt reduction in digital and paper media.	‐Process initiated to mandatory inclusion of nutrition panel including traffic light system for salt content in packaged and processed foods. ‐Plans to establish policies on taxes to discourage consumption of unhealthy food high in salt.

NA, data not available.

### 3.8 Afghanistan

Afghanistan's National Health Policy 2015–2020 and NCDs Prevention and Control Strategy 2015–2020 highlight the goal to reduce salt intake and reformulation of food products.^66^ Afghanistan developed the food‐based dietary guidelines (FBDGs) in 2015 and established a recommended daily salt intake of < 5 g/day.^67^

### 3.9 Bangladesh

Bangladesh highlighted some salt reduction measures in the Multisectoral Action Plan for Prevention and Control of NCDs (2018‐2025), such as reformulation of food products, nutritional labeling for all pre‐packaged foods, discouraging sale of processed foods high in salt in schools and work place catering facilities and mass media campaigns.^68^ FBDGs were developed in 2000 and revised in 2013.^69^

### 3.10 Bhutan

Bhutan previously set a goal of a 15% reduction in mean salt intake by 2020.^70^, ^71^ Information on whether these targets were achieved, to the best of our knowledge, has not yet been published in the public domain. Specific in‐country strategies have included setting recommended salt intake levels, collaboration with food industries to limit the salt contents in processed foods and restriction in importing high salted processed foods.^70^ FBDGs were published in 2011.^70^

### 3.11 India

India commenced working on a salt reduction program over a decade ago^18^; however, a major milestone started only after the initiation of the project “Developing the evidence base for a national salt reduction program for India” in 2014.^72^ This was a 3‐year research‐based project which aimed to generate and provide data to develop a culturally tailored national salt reduction program in India.^72^ In line with the WHO's Global Action Plan for the prevention and control of NCDs (2013‐2020), India developed a National Action Plan and Monitoring Framework for Prevention and Control of NCDs in 2013, which set a 20% relative reduction of mean population salt intake by 2020 and subsequently 30% by 2025.^73^ India's Multisectoral Action Plan for Prevention and Control of NCDs (2017‐2022) also included a range of salt reduction measures.^74^ These included attempts at a regulator reform for the reformulation of processed foods to limit the amount of salt, promote low salted healthy food in trains and at railway stations, and the introduction of a food tax on high in salt items.^74^ FBDGs lunched in 1998 and revised in 2011.^75^

### 3.12 Maldives

Maldives has incorporated some salt reduction measures in its Multisectoral Action Plan for the Prevention and Control of NCDs (2014‐2020).^76^ Proposed strategies included developing and implementation of national salt reduction strategies in line with WHO recommendations; regulate and advocate private industry to voluntarily reduce salt in packaged food and monitor compliance; public health campaigns through mass and social media to encourage consumers to eat less salt; promote nutrition labelling according to international standards for all pre‐packaged foods; testing and monitoring of salt content in processed food; the banning of high‐sodium foods in schools.^76^ FBDGs guidelines were published in 2018.^77^

### 3.13 Nepal

Despite the associated health and economic benefits, there has not been any specific salt reduction programs in place in Nepal thus far.^18^ However, plans and strategies have been incorporated in Multisectoral Action Plan for the Prevention and Control of NCDs (2014‐2020) to initiate salt reduction activities.^78^ Strategies included were ‐ baseline assessment of salt intake, develop and implement national salt strategies in line with WHO recommendations. These included attempts to regulate salt content in packaged food and monitor compliance, banning of foods high in salt from school premises and workplace catering facilities, and develop advocacy and awareness programs to educate communities regarding the potentially harmful effects of excessive salt use through various media such as health camps, articles in newspapers, interviews on television and radio, press meetings.^78^ Nepal has started collecting data on NCDs risk factors since 2003; however, salt‐related information has only been included from 2013.^48^ FBDGs were developed in 2004 and revised in 2012.^79^

### 3.14 Pakistan

In Pakistan, the Punjab Pure Food Rules 2011 and Sindh Food Regulations 2018 set food target salt levels and labelling guidelines.^66^ Dietary guidelines for better nutrition were developed in 2018 that set the recommended daily salt intake of < 5 g/day, aiming to reduce salt intake in the general population.^80^

### 3.15 Sri Lanka

Sri Lanka established a National Multisectoral Action Plan for the Prevention and Control of NCDs (2016‐2020), which proposed to develop a national salt reduction strategy with a goal of 10% relative reduction in mean population salt/sodium intake by 2020, and subsequently a 30% relative reduction by 2025.^81^ Interestingly that there was an interest to conduct urinary salt surveys on a rolling basis of every 3 years, and cross‐sectional surveys to determine the main sources of sodium. This was thought to allow both evaluation and locally tailored salt reduction interventions. Other novel trials included food labeling and a traffic light system for salt contents, establish policies on taxes to discourage consumption of unhealthy food high in salt.^71^, ^81^, ^82^ FBDGs were first lunched in 2002 and revised version were published in 2011 and in 2016.^83^

## 4 DISCUSSION

To the best of our knowledge, we have presented the first systematic review synthesizing available information on factors influencing salt intake and current salt reduction initiatives in South Asian countries. Salt intake in South Asian countries ranged from 4.4 g to 17 g/day, averaging 10 g/day, which is twice WHO recommended level of < 5 g/day. This intake level is comparable to findings of previous reviews conducted in Southeast Asia (SEA) which reported nearly double than WHO recommended salt intake.^84^, ^85^ The highest and lowest intake levels, in one review, were reported to be 18.08  and 0.40 g/day in Thailand and Indonesia, respectively.^85^ In another review, the highest intake was reported to be 9.98 g/day in Vietnam and lowest to be 6.52 g/day in Malaysia.^84^ Similarly, the International Study of Macro‐/Micro‐nutrients and Blood Pressure (INTERMAP Study) reported comparable findings of daily salt intake from Japan (11.6±3.3 g), China (13.3±5.9 g), the United Kingdom (8.5±2.9 g), and the United States (9.5±3.5 g) with a mean intake of 10.6±4.2 g/day.^86^ Likewise, nationally representative study of Korea conducted in 2009 to 2011 reported the mean salt intake among adults to be 10.99 g/day.^87^ Similar salt intake findings were reported from a systematic review and meta‐analysis conducted in Australia, that is, 9.6 g/day.^88^

The measurement of salt/sodium intake at a population level is the initial step for developing and monitoring activities targeting salt reduction programs; however, in many South Asian countries, lack of baseline measures of salt intake at country level has hindered the development, implementation and effective evaluation of sodium reduction programs. Twenty‐four hour urinary sodium excretion is considered the gold standard method due to its accuracy and avoidance of reporting biases.^89^ However, it may not be feasible in low‐resources settings, especially in a large sample at a national level due to its high associated cost, lack of skills and competencies, low priority of the salt‐reduction program, and lack of political support in LMICs.^89^, ^90^ Furthermore, under‐ or over‐collection of urine, daily intra‐individual variability of sodium intake, not accounting of sodium loss by sweat and feces, and use of diuretics may result in over‐ or under‐estimation of true sodium intake.^46^ For example, diuretics diminish sodium reabsorption in different segments of the renal tubular system, resulting in an increase in urinary sodium and water excretion.^91^ Alternatively, the validated spot urine method may be applied in settings where the 24‐h urinary sodium method is not feasible together with dietary survey to measure both sodium intake and the food sources of sodium.^90^ Several methods of estimating 24‐h urine sodium excretion from spot urine have been developed and applied^92^, ^93^, ^94^, ^95^; however, its accuracy, validity, and reliability are still questionable. Future studies are suggested to validate the spot urine method to predict 24‐h sodium excretion in resource limited community settings of South Asian countries.

South Asian countries have faced continuous challenges in developing and implementing salt reduction programs. The challenges include the availability of limited data on salt intake, sources of dietary salt remain difficult to define across food groups, lack of nutritional databases and food source tables of local and processed foods, lack of knowledge and behavior related to salt intake, and economic analysis of salt reduction programs at both at country and regional level.^96^ Further, lack of support from food companies and salt industries, and lack of political support will hinder the salt reduction program.^18^, ^96^ Despite the data scarcity on salt intake across the region, it should not hinder the initiation of salt reduction programs to prevent the burden of hypertension and CVDs.^97^

While consumer food groups containing salt have proven difficult to determine, the likely major source of salt intake in the South Asian region is discretionary salt use during cooking and/or at the table. Similar results have been reported from some other Asian countries. Systematic reviews conducted in SEA reported that discretionary salt use during cooking and/or at the table was the major source of salt intake in this region.^84^, ^85^ For instance, 98% of respondents cooked with salt in Vietnam,^80^ 83% of respondents always added salt or salty sauce to foods during cooking in Malaysia,^80^ 58% sodium is attributed to added salt in Phillipines,^80^ and 14–56% sodium can be attributed to added salt in Indonesia.^79^ Similarly, INTERMAP study reported that 76% and 9.5% of sodium was attributed from salt added during cooking at home in China and Japan, respectively.^47^ The corresponding data of the United Kingdom and the United States were 5% and 29% (overestimated), respectively.^47^ We suggest that future studies include related indicators to measure the sources of sodium in diet at the meal table in community settings.

Salt reduction approaches should be tailored to the local context, taking into account the differences in food and life‐style related behaviours,^10^ including the nature of salt intake.^46^ In HICs, the majority of salt comes from processed foods, therefore salt reduction approaches have been focused primarily on processed food; whereas in the LMICs, a community‐based approach would be more feasible and effective because the main source of salt is discretionary either as added during cooking and/or at the table.^46^, ^98^, ^99^, ^100^, ^101^ Elements of community engagement are critical to engage community members at the formative stage to explore social and cultural drivers of high salt intake in food.^60^, ^102^ Co‐designing salt reduction interventions with community members based on formative research can harness the community's support, enthusiasm, and lead to sustainable reductions in salt intake.^60^, ^102^ For instance, community‐based intervention of Vietnam and Australia were able to achieve approximately 5% and 10% reduction in salt intake between baseline and follow‐up.^60^, ^61^

However, the gradual increment in consumption of processed foods high in salt in LMICs including South Asia suggests a changing food and lifestyle related behaviour,^103^ and reiterates the need for formative research to design community‐based interventions. STEPS surveys of Nepal (2013 and 2020) reported that consumption of processed foods high in salt increased from 11.5% to 19.5% between two survey periods.^38^, ^48^ Corresponding data for Bangladesh (13.5% in 2018),^29^ Bhutan (11.5% in 2020),^30^ Sri Lanka (26.6% in 2015),^50^ and Afghanistan (12.1% in 2018),^15^ were similar. Thus, culturally tailored integrated policy including community‐based awareness raising program to encourage people to use less salt; voluntary and mandatory labeling of sodium contents, reformulation and limiting sodium in processed and junk foods both in imported and in‐country production (including sodium regulation in informal food sale such as street foods) are some recommended strategies that can reduce population salt intake. Importantly, political will and multi‐stakeholder engagement at all levels should mandate collaborative work with food industries and food vendors to achieve the goal of 30% reduction in mean salt intake by 2025. Further salt‐related research and studies are necessary at national and regional level in collaboration with regional level stakeholders and WHO.

A limitation of our study is that despite extensive search through databases, we could not find relevant articles, reports and data on salt intake, sources of salt, and salt reduction initiative for some South Asian countries. We are also cautious with interpretation of our findings as some of the selected studies were heterogeneous with respect to sodium estimation and low sample size. Indeed, the methods used for sodium estimation varied between studies, we acknowledge there are many gaps where sodium levels are difficult to determine without the use of 24‐h urine sampling. Given the heterogeneity in methodology of the selected studies, data were not pooled further for meta‐analysis. On the other hand, our aims were to conduct a narrative synthesis.

## 5 CONCLUSION

Available evidence suggests that South Asian countries have moderate to high levels of salt intake based on the limited evidence available and random urine sampling for excreted sodium levels. Salt reduction initiatives in most South Asian countries are still in the planning phase and are yet to be fully implemented or outcomes have not been evaluated or reported. While planning is aligned with the WHO's three pillars for salt reduction strategies, evidence is lacking of outcome measures. Scaling up community‐wide salt reduction strategies is imperative for reducing salt intakes in this region. Finally, there is the appreciation that without community literacy, it is likely salt addition during in home food preparation (cooking) as well as the use of discretionary table salt will continue to contribute to excess salt use in these Asian countries. More studies are needed to test the effectiveness and scalability of various strategies discussed in this review.

## CONFLICT OF INTERESTS

The authors report no conflicts of interest to disclose.

## AUTHOR CONTRIBUTIONS

KG and SRM conceived the study. KG wrote the first draft of the manuscript. KG and GS conducted the database search and literature review. KG, SRM and GS performed the analysis and interpretation. KG, SRM, GS, DN, AS, RP, PK, CSM revised the manuscript. All the authors approved the final version of the manuscript.

## Supporting information

Supporting material

Supporting material
