# Supplementary material for: Salt intake and salt‐reduction strategies in South Asia: From evidence to action
Source: J Clin Hypertens (Greenwich). 2021 Sep 9;23(10):1815–29. doi: 10.1111/jch.14365 (PMC8678780; doi:10.1111/jch.14365)
Supplement: Supplementary file 1 — Supporting material [file JCH-23-1815-s002.docx]

**Supplementary file 1**

Search terms used for searches in electronic databases.

Electronic databases (PubMed/Medline, Cochrane Library, Google Scholar) were searched, followed by searches in government websites to identify relevant studies through March 31, 2021. A total of 621 studies were screened, of which 21 studies were finally retained following the full text assessment.

Search terms used in PubMed/Medline and Cochrane Library with modification of same in Google Scholar: (Sodium Chloride OR urinary sodium OR salt) AND (intake OR ingest* OR eat* diet* consume* excrete* AND (South Asia) AND Nepal OR India OR Bangladesh OR Bhutan OR Sri Lanka OR Maldives OR Afghanistan OR Pakistan OR SAARC) AND (hypertension OR blood pressure OR micronutrients OR cardiovascular disease OR stroke)
